# Postoperative drainage management and wound complications following resection of lower limb soft tissue tumors: a retrospective cohort study

**DOI:** 10.1007/s00423-023-02939-9

**Published:** 2023-05-20

**Authors:** A. L. H. Gerken, P. Jawny, H. Weigl, C. Yang, J. Hardt, F. Menge, P. Hohenberger, C. Weiß, C. Reißfelder, J. Jakob

**Affiliations:** 1 Department of Surgery, University Medical Center Mannheim, Medical Faculty Mannheim, Heidelberg University, Mannheim, Germany; 2 Department of Cardiothoracic Surgery, University Hospital Augsburg, Medical Faculty Augsburg, Augsburg University, Augsburg, Germany; 3 Sarcoma Unit, Department of Surgery, University Medical Center Mannheim, Medical Faculty Mannheim, Heidelberg University, Theodor Kutzer-Ufer 1-3, 68167 Mannheim, Germany; 4 Department of Biometry and Statistics, University Medical Center Mannheim, Medical Faculty Mannheim, Heidelberg University, Mannheim, Germany; 5 DKFZ-Hector Cancer Institute, Medical Faculty Mannheim, Heidelberg University, Mannheim, Germany

**Keywords:** Postoperative wound complications, Soft tissue tumors, Sarcoma, Drainage management, Definition

## Abstract

**Purpose:**

Postoperative wound complications are common in patients undergoing resection of lower extremity soft tissue tumors. Postoperative drainage therapy ensures adequate wound healing but may delay or complicate it. The aim of this study is to evaluate the incidence of postoperative wound complications and delayed or prolonged drainage treatment and to propose a standardized definition and severity grading of complex postoperative courses.

**Methods:**

A monocentric retrospective analysis of 80 patients who had undergone primary resection of lower extremity soft tissue tumors was performed. A new classification was developed, which takes into account postoperative drainage characteristics and wound complications. Based on this classification, risk factors and the prognostic value of daily drainage volumes were evaluated.

**Results:**

According to this new definition, regular postoperative course grade 0 (no wound complication and timely drainage removal) occurred in 26 patients (32.5%), grade A (minor wound complications or delayed drainage removal) in 12 (15.0%), grade B (major wound complication or prolonged drainage therapy) in 31 (38.8%), and grade C (reoperation) in 11 (13.7%) patients. Tumor-specific characteristics, such as tumor size (p = 0.0004), proximal tumor location (p = 0.0484), and tumor depth (p = 0.0138) were identified as risk factors for complex postoperative courses (grades B and C). Drainage volume on postoperative day 4 was a suitable predictor for complex courses (cutoff of 70 ml/d).

**Conclusion:**

The proposed definition incorporates wound complications and drainage management while also being clinically relevant and easy to apply. It may serve as a standardized endpoint for assessing the postoperative course after resection of lower extremity soft tissue tumors.

## Introduction

Complete surgical resection remains the central aspect of multimodal therapy for lower extremity soft tissue tumors. Adequate therapy, however, requires a prompt and complication-free transition between neoadjuvant, surgical, and adjuvant therapy [1–3]. Prolonged or complex postoperative courses due to complications such as lymphatic leakage, postoperative hemorrhage or hematoma, and wound infection are common after the resection of lower extremity soft tissue tumors and may result in wound dehiscence, functional limitations, prolonged hospital stay, and delayed adjuvant therapy. The incidence of wound complications reported in these cases ranges from 17.6% to 48% [4, 5]. The high variability in these results may be explained by heterogeneous definitions [6]. Nevertheless, there is a certain consensus among prior studies on defining major postoperative wound complications as the need for surgical wound repair (debridement, secondary wound closure, negative pressure wound therapy, plastic reconstructions, deep packing), for inpatient readmission for antibiotic treatment, and/or for conservative wound management including interventional procedures, such as puncture (needle aspiration) and reinsertion of wound drains, [4, 7–14].

**Fig. 1:**
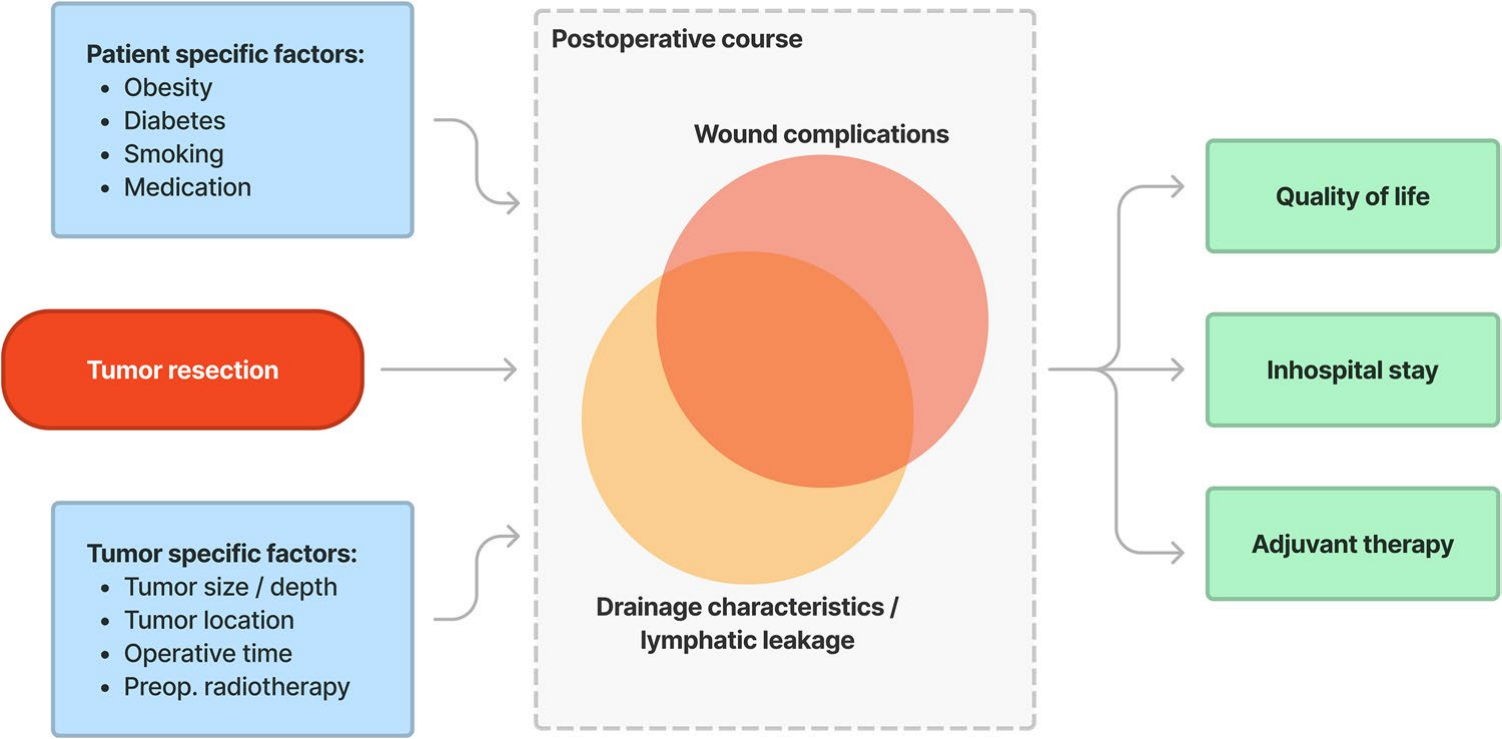
Risk factors and potential influence of wound complications after resection of soft tissue tumors on patient satisfaction, hospital stay, and oncological outcome

**Fig. 2:**
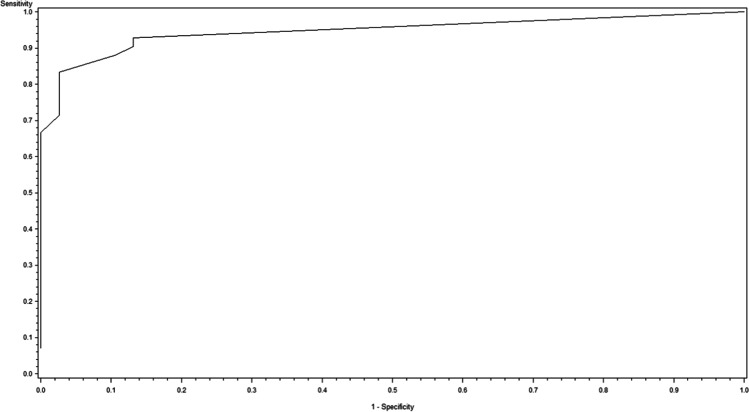
Receiver operating characteristic curve for the drainage volume on the second postoperative day as a predictor for the manifestation of a complicated postoperative wound situation grade B or C (AUC = 0.897, p < 0.0001)

Different surgical strategies are practiced to minimize postoperative wound healing disorders. These are, for example, (1) vascular and plastic soft tissue reconstruction, which may improve long-term outcomes but is associated with a high perioperative complication rate [15, 16], (2) the initial vacuum sealing of wounds with secondary wound closure, which can have a positive effect on wound healing [17] but also increases the risk of surgical site infections [18], and (3) intraoperative fluorescence-guided lymph vessel sealing, which might be a promising technique to prevent postoperative lymphatic leakage [19]. However, in most cases, direct wound closure with intraoperative insertion of wound drains is considered the standard of care. Under normal circumstances wound healing can be significantly improved by surgical drainage therapy during the inpatient stay. However, wound healing disorders or extended drainage therapy can also lead to prolonged hospitalization or readmissions in the context of complex courses. Currently, there is no specific and uniform guideline for postoperative wound management after the resection of lower extremity soft tissue tumors. Figure 1 illustrates the clinical relevance of wound complications and duration of drainage therapy in terms of quality of life, hospital stay, and oncological outcome and lists possible variables that influence the development of a wound healing disorder (e.g. diabetes, smoking, obesity, preoperative radiotherapy). Adequate and timely wound healing is a prerequisite for a rapid and complication-free transition from surgical to adjuvant therapy. In addition, the occurrence of wound healing disorders, just as of delayed or prolonged drainage therapy, influences patients’ quality of life [20]. The application of a specific, standardized, and uniform definition of the postoperative course, incorporating a complete and accurate documentation of postoperative wound complications and drainage therapy, is an important factor in enabling an objective evaluation and comparison of the postoperative outcomes in different studies. Based on such a definition, further diagnostic and therapeutic options can be evaluated to avoid relevant wound complications in the future and to allow safe outpatient management Fig. 2.

The aim of this study is, therefore, to retrospectively quantify and objectively evaluate postoperative drainage therapy and the incidence of postoperative wound complications after primary resection of soft tissue tumors of the lower extremity. Based on these data, a simple and reliable clinical measure for the early detection of prolonged or complex courses will be established. This will primarily serve to improve patient care through the early identification of potential wound complications and the initiation of appropriate treatment.

## Material and methods

### Study design and population

We performed a retrospective cohort study of patients who had undergone primary resection of a soft tissue tumor of the lower extremity between April 2007 and July 2016 at the University Medical Center Mannheim. Patients with an inguinal tumor and those who had undergone a two-stage resection after an external primary resection were not included. Patients in the study period were identified retrospectively via a prospective electronic database maintained by the sarcoma unit. The study was conducted in accordance with the Declaration of Helsinki and approved in advance by the responsible ethics committee of the Medical Faculty Mannheim of the University of Heidelberg (ID 2019-832R).

### Data acquisition

Electronic patient data and paper-based patient records were used for data extraction. General patient data, tumor characteristics, data regarding the surgical procedure, the daily discharge of intraoperatively inserted drains and their postoperative management, as well as postoperative morbidity and the need for postoperative interventions or reoperations were collected. Depending on availability, records of the primary stay and subsequent outpatient controls were taken into consideration. Data were fully anonymized after the review of patient records and subsequently processed.

### Surgical procedures

The aim of the surgical interventions performed was always complete tumor resection in healthy tissue while preserving the limb. Wide resections were performed with a safety distance from the resection margin to the tumor of at least 2 cm in the longitudinal and transverse axis and at least 1 cm in depth. Depending on the local findings and infiltration of anatomical boundary structures, the procedure was extended intraoperatively to a compartment resection to achieve a tumor-free resection margin, or reduced to a marginal resection to ensure adequate functional preservation. Following complete resection, suction drains (Redon drain, e.g., ORIFLEX V 600 ml, Oriplast Krayer GmbH, Neunkirchen, Germany) were placed in the wound cavity if necessary, depending on the extent of the procedure, the local wound situation, the tumor location, and histologic subtype. Drain placement was more restrictive in less infiltrative tumors such as atypical lipomatous tumors which may be treated by marginal resection (e.g. compared to myxofibrosarcoma or undifferenciated pleomorphic saromcas), because less bleeding and wound complications were expected. Wound closure was usually performed using subcutaneous single button sutures. The cutis was closed with single button sutures using the Donati suture technique or skin staples.

### Classification of postoperative wound complications (grades I-III)

Wound complications were classified into three groups according to previous studies [6, 7, 9, 10, 12–14, 21–23] and current classifications such as CTCAE (v5) [24] and Clavien Dindo [25] as shown in Table 1.

**Table 1:** Grading of postoperative wound complications according to CTCAE and Clavien Dindo for comparison with the proposed classification (compare Table 2)

Grade	CTCAE v5	Clavien Dindo	Description
Grade I (minor complications)	Wound complication grade I Wound infection grade I Wound dehiscence grade I	≤ grade II	Wound dehiscence, wound effusion, seroma or hematoma in the surgical area **without** indication for interventional or surgical treatment
Grade II (major complications)	Wound complication grade II Wound infection grade II Wound dehiscence grade II	grade IIIa	Appropriate complications with indication for **interventional treatment** by means of: • Puncture or secondary drainage installation • Inpatient readmission for local wound therapy, or intravenous antibiotic therapy
Grade III (reoperation)	Wound complication grade III Wound infection grade III Wound dehiscence grade III	grade IIIb	Need for **surgical revision** or life-threatening complication (e.g., bleeding, sepsis)

Minor complications (grade I) include the occurrence of local wound dehiscence, wound secretion, and the postoperative diagnosis of seromas or hematomas in the surgical cavity, without an indication for interventional or surgical treatment. They thus correspond to Clavien Dindo grade II, CTCAE wound complication grade I, wound infection grade I, and wound dehiscence grade I.

Major complications (grade II) were defined as corresponding complications with an indication for interventional treatment by puncture or secondary drainage, hospital readmission for local wound therapy, or intravenous antibiotic therapy. They thus correspond to Clavien Dindo grade IIIa, and CTCAE wound complication grade II, wound infection grade II, and wound dehiscence grade II.

In addition, severe wound complications in need of surgical revision or exhibiting life-threatening complications were defined as grade III, which corresponds to Clavien Dindo grade IIIb and above, and CTCAE wound complication grade III, wound infection grade III, and wound dehiscence grade III and above.

### Drainage management specifications

The intraoperative placement and postoperative management of wound drains after resection of lower extremity soft tissue tumors were not standardized during the study period. The indication for intraoperative drainage and the corresponding configuration were patient-specific, depending on the extent of resection, the intraoperative wound situation, and the tumor location. Postoperatively, the drainage output was recorded daily. The internal hospital guideline for safe drainage removal was an output of less than 50 ml/24 h. In the case of irritation-free wound conditions but delayed or even prolonged wound secretion via the drainage, patients were discharged, and an outpatient continuation of the drainage therapy was implemented in individual cases.

### Postoperative course definition and severity grading (grades A-C)

Based on clinical considerations and in accordance with previously published classifications [26], we developed a specific, standardized, and uniform definition of the postoperative course that incorporates postoperative drainage details into the preexisting definitions of wound complications as defined above. Drainage therapy was divided into three groups. Removal of intraoperatively placed drains by the fourth postoperative day with a daily discharge of less than 50 ml was defined as regular drainage therapy. Delayed wound drainage was defined as persistent secretion via the drains for more than four and less than 10 days. Extended wound drainage was defined as persistent secretion for at least 10 days postoperatively. Both definitions of postoperative wound complications and drainage details were merged into a new classification of the postoperative course after resection of lower extremity soft tissue tumors, which was divided into four groups as shown in Table 2. A regular postoperative course (grade 0) was defined as timely removal of the surgically inserted drains without the occurrence of relevant wound complications. A delayed postoperative course (grade A) was defined as delayed wound drainage and/or the occurrence of minor wound complications without the need for intervention. A complex postoperative course (grade B) was defined as one in which extended wound drainage and/or the occurrence of major wound complications occurred and were an indication for intervention. Ultimately, the need for a secondary operation due to wound complications was defined as grade C. All the patients were assigned to one of these four groups.

**Table 2:** Extended definition and severity grading of the postoperative course after resection of soft tissue tumors reflecting the patient’s burden by merging postoperative wound drainage management and complications

Grade	Drainage therapy	Wound complications	Description
0	In time	No complications	Timely drainage removal within 4 days, no wound complication
A	Delayed	Minor complications*	Delayed wound drainage ≥ 5th postoperative day and/or wound complications without need for intervention
B	Extended	Major complications**	Prolonged wound drainage ≥ 10th postoperative day and/or wound complications necessitating intervention (e.g. puncture/drainage of seroma, readmission for wound therapy or intravenous antibiotic therapy)
C		Reoperation***	Need for reoperation (e.g., for bleeding or wound breakdown)

^*^minor: CTCAE Grade I, Clavien Dindo ≤ grade II; **major: CTCAE grade II, Clavien Dindo ≤ grade IIIa; ***; reoperation: CTCAE grade III, Clavien Dindo ≤ grade IIIb (compare Table 1)

### Statistical analysis

All statistical calculations were performed using SAS, release 9.4 (SAS Institute Inc., North Carolina, USA). For qualitative data, absolute and relative frequencies were estimated. For quantitative and approximately normally distributed data, mean values and standard deviations were calculated. For ordinally scaled or skewed variables, the median value along with range was considered instead. In order to compare two groups regarding a categorical factor, the Chi^2^ test was applied. Only if the preconditions of the Chi2 test were not fulfilled (under the null hypothesis expected frequencies less than 5), Fisher's exact test was used instead. For ordinally scaled variables, the trend test by Cochran Armitage was performed. The mean values of a quantitative variable were compared with a 2 sample t test, if the data were approximately normally distributed. Otherwise, Wilcoxon 2 sample test was used as a location test.

For each postoperative day, a univariate logistic regression analysis was performed to evaluate associations between a binary outcome (i.e. complex postoperative course) and drainage volume on that day as an independent variable. The area under the ROC curve (AUC) was used to quantify the predictive power of the relevant statistical model in order to identify the most appropriate day for predicting the outcome. Odds ratios were assessed together with 95% confidence intervals. Furthermore, the optimal cutoff at which the Youden index (sensitivity + specificity—1) is maximal was estimated.

The results of the calculations were considered statistically significant if a p value < 0.05 was obtained.

## Results

### Demographics

Our study population consisted of 80 patients who underwent primary resection of a soft tissue tumor of the lower extremity. Of these, 53 (66.3%) were men and 27 (33.7%) were women. The mean age was 60.2 (± 14.4) years. The most common histologic subtype was liposarcoma, which was observed in 32 cases (40.0%). The mean tumor size was 11.7 (± 5.6) cm. Seventeen patients (21.3%) received neoadjuvant radiotherapy, and three patients (3.8%) received intraoperative radiotherapy. Neoadjuvant chemotherapy was performed in 20 (25.0%) patients, while 12 patients (5.0%) were treated neoadjuvantly with isolated limb perfusion (ILP). Additional patient, tumor, and treatment characteristics are shown in Table 3.

**Table 3:** Patient and tumor characteristics (n = 80). The data is presented as absolute and relative values (percentages), mean ± standard deviation, or median together with range

Patient characteristics	
Sex (♀:♂)	27 (33.7%); 53 (66.3%)
Age (years)	60.2 ± 14.4
BMI (kg/m^2^)	27.1 ± 4.8
Obesity	19 (23.8%)
Diabetes	8 (10.0%)
Smoking	6 (7.5%)
Aspirin therapy	14 (18.5%)
Inpatient details	
Duration of hospital stay (days)	11 (1—83)
Duration of surgery (min)	108 (20—475)
Drainage details	
Duration of drainage (days)	6 (0—67)
< 5 days	32 (42.1%)
5–10 days	10 (13.2%)
> 10 days	34 (44.7%)
Tumor entities	
Liposarcoma	32 (40.0%)
Sarcoma NOS	13 (16.2%)
Lipoma	12 (15.0%)
Fibrosarcoma	8 (10.0%)
Leiomyosarcoma	3 (3.8%)
MPNST	3 (3.8%)
Others	9 (11.2%)
Tumor localization	
Upper thigh	66 (82.5%)
Lower thigh	14 (17.5%)
Superficial	16 (20.0%)
Deep	64 (80.0%)
Tumor size (cm)	10.5 (1.5–30)
Oncological treatment	
Neoadjuvant Radiotherapy	17 (21.3%)
Intraoperative Radiotherapy	3 (3.8%)
Neoadjuvant Chemotherapy	20 (25.0%)
Neoadjuvant ILP	12 (15.0%)

### Wound complications

The incidence of different grades of wound complications is presented in Table 4. While 38 (47.5%) patients did not develop any kind of postoperative wound complications, minor wound complications (grade I) occurred in 17 (21.3%) patients. Major wound complications involving interventional treatment (grade II) were observed in 14 (17.5%) patients. In 11 (13.7%) patients a reoperation due to wound complications (grade III) was required. Thus, in summary, 25 (31.2%) patients in our cohort developed wound healing disorders of therapeutic consequence (grades II and III), whereas 55 (68.8%) patients developed no or just minor complications (grades 0 and I). Univariate analysis between those two groups showed statistically significant differences in the operative time (p = 0.0170), postoperative albumin (p = 0.0307), duration of drainage therapy (p = 0.0001), and classification of drainage output at the time of drain removal (p = 0.0489). The detailed results are summarized in Table 5.

**Table 4:** Incidence of wound complications according to two different definitions

Conventional definition of wound complications*		Proposed definition of postoperative course**	
Grade 0	38 (47.5%)	Grade 0	26 (32.5%)
Grade I	17 (21.3%)	Grade A	12 (15.0%)
Grade II	14 (17.5%)	Grade B	31 (38.8%)
Grade III	11 (13.7%)	Grade C	11 (13.7%)

^*^definition of surgical complications according to CTCAE and Clavien-Dindo (Table 1); ** proposed new definition of postoperative course (Table 2)

**Table 5:** Comparison of risk factors for major wound complications according to two different definitions

	Conventional definition of wound complications*			Proposed definition of postoperative course**		
	Grade 0/I	Grade II/III	p-value	Grade 0/A	Grade B/C	p-value
Population (n = 80)	55 (68.8%)	25 (31.2%)		38 (47.5%)	42 (52.5%)	
Sex			0.7742 ^(1)^			0.9340 ^(1)^
male	37 (69.8%)	16 (30.2%)		25 (47.2%)	28 (52.8%)	
female	18 (66.6%)	9 (33.3%)		13 (48.2%)	14 (51.2%)	
Age	58.2 ± 14.5	64.7 ± 13.5	0.0592 ^(3)^	57.2 ± 16.0	62.9 ± 12.4	0.0767 ^(3)^
BMI	26.6 ± 4.2	28.1 ± 5.9	0.1961 ^(3)^	26.5 ± 4.2	27.6 ± 5.3	0.3457 ^(3)^
Obesity	12 (63.1%)	7 (36.8%)	0.5470 ^(1)^	8 (41.1%)	11 (57.9%)	0.5897 ^(1)^
Diabetes	5 (62.5%)	3 (37.5%)	0.7000 ^(2)^	3 (37.5%)	5 (62.5%)	0.7150 ^(2)^
Smoking	4 (66.6%)	2 (33.3%)	1.0000 ^(2)^	4 (66.7%)	2 (33.3%)	0.4159 ^(2)^
Albumin						
preoperative	37.7 ± 5.2	37.9 ± 3.3	0.8895 ^(3)^	38.1 ± 4.8	37.3 ± 4.8	0.5754 ^(3)^
postoperative	31.5 ± 4.8	28.5 ± 4.1	**0.0307** ^(3)^	32.6 ± 4.6	29.1 ± 4.4	**0.0086** ^(3)^
difference	5.5 ± 4.2	10.4 ± 4.1	**0.0045** ^(3)^	4. 8 ± 3.8	8.6 ± 4.7	**0.0123** ^(3)^
ratio	1.18 ± 0.15	1.40 ± 0.21	**0.0013** ^(3)^	1.15 ± 0.13	1.31 ± 0.21	**0.0074** ^(3)^
operative time	100 (20–324)	122 (30–475)	**0.0170** ^(4)^	74 (20–270)	130 (30–475)	** < 0.0001**^(4)^
Tumor size	10 (1.5–22)	11 (6–30)	0.1530 ^(4)^	8.5 (1.5–20)	12.5 (6–30)	**0.0004** ^(4)^
Tumor location			0.2052 ^(1)^			**0.0484** ^(1)^
upper thigh	43 (65.2%)	23 (34.8%)		28 (42.4%)	38 (57.6%)	
lower thigh	12 (85.7%)	2 (14.3%)		10 (71.4%)	4 (28.6%)	
Tumor depth (n[%])			0.2278 ^(2)^			**0.0138** ^(1)^
suprafascial	13 (81.3%)	3 (18.7%)		12 (75.0%)	4 (25.0%)	
infrafascial	42 (65.6%)	22 (34.4%)		26 (40.6%)	38 (59.4%)	
Preop. RTx	13 (65.0%)	7 (35.0%)	0.6761 ^(1)^	7 (35.0%)	13 (65.0%)	0.1961 ^(1)^
Preop. CTx	14 (70.0%)	6 (30.0%)	0.8892 ^(1)^	7 (35.0%)	13 (65.0%)	0.1961 ^(1)^
Drainage details						
Duration of drainage (days)			**0.0001** ^(5)^			** < 0.0001**^(5)^
< 5 days	30 (93.8%)	2 (6.2)		30 (93.8%)	2 (6.2)	
5–10 days	5 (50%)	5 (50%)		5 (50%)	5 (50%)	
> 10 days	17 (50%)	17 (50%)		0	34 (100%)	
Drainage output at removal			**0.0489** ^(5)^			0.1946 ^(5)^
< 50 ml/d	34 (70.8%)	14 (29.2%)		22 (45.8%)	26 (54.2%)	
50-100 ml/d	7 (70.0%)	3 (30.0%)		3 (30.0%)	7 (70.0%)	
> 100 ml/d	4 (36.4%)	7 (63.6%)		3 (27.3%)	8 (72.7%)	
Discharge with lying drainage	6 (54.5%)	5 (45.5%)	0.3085 ^(2)^	0 (0.0%)	11 (100.0%)	**0.0009** ^(1)^

^*^definition of surgical complications according to CTCAE and Clavien-Dindo (Table 1); ** proposed new definition of postoperative course (Table 2); CTx, chemotherapy; RTx, radiotherapy. Tests: ^1^Chi2 test; ^2^Fischer's exact test; ^3^t test; ^4^Wilcoxon 2 sample test; ^5^Cochran Armitage trend test. Significant *p*-values are printed in bold

### Drainage management

Intraoperative drain placement was not indicated in seven patients (8.8%), while 48 patients (60.0%) were treated with a single and 21 patients (26.3%) with two suction drains. In four (5.0%) patients, because the large resection area was an indication for plastic reconstruction, negative pressure wound therapy (NPWT) was performed as a bridging procedure until a completely tumor-free resection margin could be confirmed pathologically. Timely drain removal was achieved in 32 out of 76 cases (42.1%). In a total of 10 patients (13.2%), drain removal was delayed between postoperative days 5 and 10. Drainage therapy was prolonged by more than 10 days in 34 cases (44.7%). Due to persistently high daily drainage volumes, 11 patients (14.5%) were discharged with an indwelling wound drain. Drainage removal was then performed either by the patient’s primary care physician or as part of an outpatient appointment as soon as drainage output was less than 50 ml/d. This liberal outpatient drainage management proved to be safe in our cohort. None of the patients discharged with drainage presented bleeding, sepsis, or major wound complications as an indication for surgical revision.

### Lymphatic morbidity and reoperations

A postoperative seroma/lymphocele was documented in 29 patients (36.3%), and 18 patients (22.5%) required a needle aspiration or secondary drainage of the seroma. The incidence of wound infections was 13.8%. Eight patients (10.0%) required intravenous administration of antibiotics. In 11 patients (13.8%), a reoperation had to be performed after conservative management failed, mainly for surgical wound revision and reinsertion of a suction drain (n = 5), followed by NPWT (n = 4) and wound revision with secondary suture of the skin (n = 2).

### Comparison of postoperative courses

The incidence of different grades of the proposed extended definition and severity classification of the postoperative course is presented in Table 4. A total of 42 (52.5%) of the patients showed a complicated postoperative course, with the need for interventional or surgical care of wound healing disorders or a prolonged duration of drainage therapy of more than 10 days, corresponding to grades B or C. The comparative analyses of grades 0 and A vs. grades B and C are shown in Table 5.

### Factors influencing the postoperative course

Comparing the two groups of postoperative courses (grades 0/A vs. grades B/C), neither the patient-specific risk factors—obesity (p = 0.5897), diabetes (p = 0.7150), smoking (p = 0.4159), and preoperative aspirin use (p = 1.000)—nor neoadjuvant or intraoperative radiotherapy (p = 0.1961) were associated significantly with complex course grades B/C. However, tumor-specific and other parameters, such as tumor size (p = 0.0004), tumor location (p = 0.0484), tumor depth (p = 0.0138), and operative time (p < 0.0001) differed significantly between both groups.

### ROC analysis

Logistic regression analysis yielded a significant association between the documented daily postoperative drainage volume and the binary endpoint "complex postoperative course" (grade B or C as defined in Table 2). Drainage volume on postoperative day 4 is thus a potentially suitable predictive factor for a complex course (AUC = 0.947, p < 0.0001). This statistical model resulted in an odds ratio of 1.045 (95% CI: [1.023, 1.066]), i.e. with each ml of drainage volume the risk for a complex postoperative course increases by about 4.5%. ROC analysis delivered a threshold value of 70 ml on postoperative day 4, predicting such course with a sensitivity of 83% and a specificity of 97%.

Regarding the conventional definition for wound complications (grade 0/I vs. grade II/III as defined in Table 1), this same threshold value of 70 ml also predicted a major wound complication or reoperation with a sensitivity of 80% and a specificity of 71% (AUC = 0.815, p = 0.0002) and an odds ratio of 1.009 (CI: [1.004, 1.014]).

## Discussion

The results of this retrospective cohort study evaluating postoperative courses after the resection of lower extremity soft tissue tumors confirmed that wound complications occur frequently. The postoperative drainage volume was an independent risk factor with a predictive ability for severe complications and should therefore be included in a standardized definition of postoperative course.

### Definition of wound complications after resection of lower extremity soft tissue tumors

The incidence of wound complications after resection of lower extremity soft tissue tumors ranges from 17.6% to 48% with an overall wound complication rate of 30.2% (95% CI 26.56–33.47) as published in a recent systematic review and meta-analysis of 18 studies [6]. The authors, however, noted a large heterogeneity, most likely due to inconsistent definitions of major wound complications. Most of the previously published studies refer to the RCT by O’Sullivan et al., defining major wound complications as the need for reoperation or nonsurgical wound management, including interventional procedures such as needle aspiration of seroma or readmission for intravenous antibiotic therapy [7]. Some studies do not regard the necessity for intravenous antibiotic treatment alone as a major wound complication [4, 12]. To further standardize a possibly clinically more appropriate definition, we merged the definition by O’Sullivan et al. with generally accepted definitions and severity gradings of wound healing disorders, namely Clavien Dindo [25] and CTCAE (v5) [24]. The incidence of major wound complications in our cohort on applying this merged definition was comparable to the results of previous studies. Interestingly, the amount of wound secretion was associated with the incidence of major complications. Therefore, lymphatic leakage, such as seromas and lymphatic fistulas, seems to account for the largest proportion of postoperative wound complications in this cohort. Postoperative wound drainage is an effective treatment in this regard. However, the need for prolonged drainage therapy should also be regarded as a postoperative complication because it compromises the timely transition to adjuvant therapy. A previously published RCT evaluation of seroma output after femoral lymph node dissection stated that patients were willing to pay money to decrease the period of drainage therapy by 4 days [20]. In this regard, the nationwide Observational Study (PROSa) demonstrated that especially patients with soft tissue tumors of the lower extremity suffer from the worst health-related quality of life postoperatively [27]. Consequently, in our opinion, prolonged drainage therapy with an increased daily drainage volume of more than 50 ml/d for more than 10 days is equivalent to a severe wound complication with needle aspiration or drain reinsertion and should therefore be included in the new proposal for an expanded definition as an indication for a complex course grade B. The increased incidence of serious adverse events in the postoperative course of more than 50% emphasizes the clinical relevance. Furthermore, prolonged drainage therapy commonly prevents patients from being discharged from inpatient treatment and might therefore reduce patient mobilization and satisfaction and increase treatment costs. Although discharging patients with indwelling suction drains seems to be safe with regard to major events like bleeding or infection, patients and treating physicians nevertheless commonly face logistical problems with regard to drainage supply and handling.

While the results of the present study did not confirm the patient-specific risk factors identified in previous studies [4, 28], they showed a significant association of tumor-specific measures in univariate analysis, confirming the results of previous work [4, 29], particularly after having applied our revised definition of postoperative courses (Table 5). In contrast to prior publications, we did not observe an association of preoperative radiotherapy with a complicated postoperative course. On the one hand, this might be due to the limited number of patients included after neoadjuvant radiotherapy. On the other hand, it might be argued that a reduction in the postoperative complications associated with lymphatic leakage was due to a sealing effect of radiotherapy on lymphatic vessels. Interestingly, patients with complex postoperative courses showed a more pronounced postoperative drop of albumin here. Preoperative high protein nutrition might be able to reduce this postoperative decrease. This aspect should be addressed in further trials.

Daily drainage output was identified as a reliable predictor for major wound complications and complex or prolonged courses (grade B or C). Although no specific therapeutic approaches for early intervention were evaluated in this study, the proposed combined definition of postoperative drainage volume and wound complications could simplify clinical management in the future and allow for individual risk stratification and early detection of potentially complicated wound situations. The definition might be suitable as a standardized endpoint for further studies. The predictive value of daily postoperative drainage output has a potential relevance for clinical decisions and guidelines. It can be used to more accurately educate patients about individual risks and allow for better planning of postoperative care, including early and safe transition to outpatient management. In patients with a postoperative drainage output above the threshold on postoperative day 4, early interventions (e.g., instillation, sclerotherapy, radiation therapy), prolonged drainage therapy, or surgical wound revision should be considered early.

### Drainage management recommendations

Based on our data, we have subsequently implemented the following standard for postoperative care after resection of lower extremity soft tissue tumors at our department. In general, intraoperative placement of at least one wound drain is performed. In individual cases, e.g., superficial resections and acceptable tumor size, drainage may not be necessary at all. Drainage removal should be performed at low flow rates (i.e. < 50 ml/d or < 30 ml/d, depending on the localization), no later than postoperative day 4. If a delayed, but uncomplicated postoperative course (grade A) can be assumed based at a flow rate lower than 70 ml/d on day 4, discharge and outpatient monitoring by the primary care physician can be implemented after prior patient training. This outpatient approach has been shown to be feasible and safe. However, if there are indicators of a prolonged or complex drainage course (grades B or C), further diagnostics and, if necessary, early therapeutic intervention should be considered.

### Limitations

Because of the monocentric and retrospective design of our study, the results are subject to the expected limitations. The documentation of daily drainage volumes was standard operating procedure in our department. Data collection included the relevant risk factors and known manifestations of postoperative wound healing disorders. However, other factors could also be important. Only univariate analyses have been performed, since we focused on the association between drainage characteristics and wound complications and not on a general identification of risk factors. Further prospective studies need to minimize bias, such as by using larger sample sizes, by identifying further variables and potential risk factors, and by adding multivariate analyses to test for the simultaneous influence of different parameters. Our study population is comparable to those in other recent studies investigating wound complications after the resection of soft tissue tumors that focus exclusively on the lower extremity. Our data concerning the parameters of the definition allowed us to detect significant differences in daily drainage volumes and define a cutoff for the prediction of a complex or prolonged postoperative course.

### Future perspectives

Through adequate documentation and evaluation of postoperative drainage volume, possibly through the widespread use of digital drainage assessment tools and the application of a standardized definition, it may become possible to compare complication rates in the future. However, further studies, ideally prospective ones, are needed to validate the proposed definition. These studies might also consider the Comprehensive Complication Index (CCI), as it is a different tool reflecting the gravity of the overall complication burden on the patient that has been developed for RCTs. In addition to the cutoff for safe drainage removal, the safety and efficacy of postdischarge outpatient wound management with continued drainage should be investigated. Furthermore, this will create the prerequisites for future studies for evaluating how prolonged or complex postoperative courses can be avoided or efficiently treated.

## Conclusion

Based on this retrospective analysis of 80 patients and the current literature, we propose a specific and uniform definition for the evaluation of the postoperative course after the resection of lower extremity soft tissue tumors as described in Table 2. The proposed definition strengthens the ability to identify and compare postoperative wound healing disorders as well as prolonged drainage therapies, making it a helpful tool for clinical decision making regarding postoperative follow-up, as well as for improving the comparability of future studies. Our data indicate that a drainage volume greater than 70 ml/d on postoperative day 4 is a possible indicator for the manifestation of a prolonged or complex postoperative course (grade B or C).
